# Effect of idebenone supplementation in a semen extender on boar spermatozoa quality during liquid storage

**DOI:** 10.14202/vetworld.2025.1479-1486

**Published:** 2025-06-10

**Authors:** Rehardus Ricco Pantecostoma, Jatesada Jiwakanon, Saksiri Sirisathien

**Affiliations:** 1 Veterinary Science Program, Faculty of Veterinary Medicine, Khon Kaen University, Khon Kaen 40002, Thailand; 2 Research Group for Animal Health Technology, Faculty of Veterinary Medicine, Khon Kaen University, Khon Kaen 40002, Thailand; 3 Division of Theriogenology, Faculty of Veterinary Medicine, Khon Kaen University, Khon Kaen 40002, Thailand

**Keywords:** antioxidant, artificial insemination, boar semen, idebenone, lipid peroxidation, oxidative stress, sperm preservation

## Abstract

**Background and Aim:**

In swine production, over 99% of artificial insemination (AI) procedures utilize boar semen preserved in liquid form at 17°C for up to 5 days. However, spermatozoa are highly susceptible to oxidative stress during storage, which impairs motility, membrane integrity, and overall fertility. Reactive oxygen species-induced lipid peroxidation (LPO) compromises sperm structure and function. Although antioxidants are used to mitigate oxidative damage, idebenone (IDB) – a short-chain benzoquinone with potent mitochondrial antioxidant properties – has not been studied in boar semen preservation. This study aimed to investigate the effects of IDB supplementation in a semen extender on boar spermatozoa quality during 120 h of liquid storage at 17°C.

**Materials and Methods:**

Ejaculates from 25 Duroc boars were diluted with Beltsville Thawing Solution to a final concentration of 30 × 10^6^ sperm/mL. In Experiment 1, semen samples (n = 13) were treated with 0, 78, 156, 312, 625, or 1250 nM of IDB and assessed at 24-h intervals for motility (computer-assisted sperm analysis), viability (eosin-nigrosin), and membrane integrity (hypo-osmotic swelling test). In Experiment 2, 78 nM IDB was selected for its optimal effects and further tested (n = 12) for acrosome integrity (fluorescein isothiocyanate-conjugated peanut agglutinin), capacitation status (chlortetracycline staining), LPO (thiobarbituric acid reactive substances), and kinematic parameters.

**Results:**

After 120 h, the 78 nM IDB group showed significantly improved total motility (81.4%) and membrane integrity (60.3%) compared to control (72.9% and 46.7%, respectively; p < 0.05). Viability was higher in IDB-treated groups, and high concentrations (625–1250 nM) reduced motility. In Experiment 2, 78 nM IDB improved total and progressive motility (84.0% and 46.8%), preserved acrosome integrity (64.06% vs. 58.75%), reduced capacitated sperm (64.72% vs. 59.11%), and decreased malondialdehyde levels (51.84 nmol vs. 59.61 nmol) relative to controls (p < 0.05).

**Conclusion:**

IDB at 78 nM effectively preserves boar semen quality during 5-day liquid storage by enhancing motility, viability, membrane and acrosome integrity, and reducing oxidative stress. These findings highlight its potential as a novel antioxidant additive in AI protocols.

## INTRODUCTION

Artificial insemination (AI) in swine breeding predominantly relies on liquid-stored boar semen, which accounts for approximately 99% of AI procedures worldwide. This semen is conventionally stored at 17°C to suppress metabolic activity and extend sperm viability for up to 5 days [1, 2]. Despite this approach, a progressive decline in sperm quality occurs during storage [3, 4], primarily due to oxidative stress [5, 6]. This stress results from an imbalance between the generation of reactive oxygen species (ROS) and the antioxidant capacity of the seminal plasma. Notably, previous studies have reported increased ROS accumulation during the liquid storage of boar semen [4, 7, 8], highlighting the need for effective strategies to mitigate oxidative damage.

ROS, including superoxide (O_2_•−), hydrogen peroxide (H_2_O_2_), hydroxyl radical (OH•), and nitric oxide, are byproducts of aerobic metabolism and are predominantly generated in the mitochondria [9, 10]. While physiologically relevant concentrations of ROS are essential for processes such as sperm capacitation and oocyte interaction [11–13], elevated levels can induce detrimental effects. These include lipid membrane degradation [14–16], DNA fragmentation [17], and protein oxidation [18], ultimately impairing motility, viability, and fertilizing potential [19, 20].

A key mechanism of ROS-induced damage is lipid peroxidation (LPO), which primarily targets polyunsaturated fatty acids (PUFAs) in sperm membranes. This process is initiated when ROS extract hydrogen atoms from bis-allylic methylene groups, leading to the formation of lipid peroxyl radicals (–ROO^•^), which propagate further peroxidative reactions in a chain-like manner unless terminated by antioxidants [21, 22]. Given their high PUFA content and limited endogenous antioxidant systems, boar spermatozoa are particularly susceptible to LPO during storage [23, 24]. While antioxidant supplementation has proven effective in reducing oxidative stress in both liquid and cryopreserved boar semen [4, 8, 25, 26], the application of idebenone (IDB) – a mitochondria-targeting benzoquinone compound – has yet to be explored in this context.

IDB, a synthetic analog of coenzyme Q10 (CoQ10), features a shorter isoprenoid side chain and greater solubility, enhancing its bioavailability [27]. As an electron carrier in the mitochondrial electron transport chain, IDB can function as a potent antioxidant. Zhu *et al*. [28] have shown that CoQ10 reduces ROS levels in boar sperm, and IDB has demonstrated LPO-inhibiting effects in various tissues, including murine brain homogenates [29–33]. Although IDB has shown promise in improving sperm quality in rams [34], its potential application in boar semen preservation remains uninvestigated. The present study addresses this gap by evaluating the efficacy of IDB supplementation in preserving boar spermatozoa quality during liquid storage.

Although oxidative stress has been widely recognized as a principal factor contributing to the deterioration of boar sperm quality during liquid storage, current antioxidant strategies remain limited in their effectiveness. Most extenders rely on traditional antioxidants that may not adequately target the mitochondria – the primary site of ROS production in spermatozoa. Mitochondrial dysfunction during storage contributes to an overproduction of ROS, leading to LPO, membrane destabilization, and impaired motility. While several studies have explored the benefits of CoQ10 in ameliorating ROS-induced damage in sperm, the use of its synthetic analog, IDB, remains largely unexamined in swine reproduction. IDB is a short-chain benzoquinone with superior solubility and bioavailability compared to CoQ10, and it has demonstrated potent antioxidant activity in neural, hepatic, and reproductive tissues. Importantly, its effectiveness in preserving sperm quality has been confirmed in ruminant models, such as rams, but there are no published reports assessing its role in boar semen preservation. This represents a significant knowledge gap in swine AI technologies, particularly considering the industry’s reliance on liquid-stored semen for breeding efficiency. Therefore, the exploration of IDB as a novel antioxidant in boar semen extenders is both timely and necessary.

The primary objective of this study was to evaluate the efficacy of IDB supplementation in a commercial semen extender for preserving boar spermatozoa quality during liquid storage at 17°C over a 120-h period. Specifically, the study aimed to (i) identify the optimal concentration of IDB that supports sperm motility, viability, and membrane integrity, and (ii) assess the impact of the selected concentration on advanced sperm parameters, including acrosome integrity, capacitation status, and LPO. Through this investigation, we sought to determine whether IDB can effectively mitigate oxidative stress and prolong sperm functionality, thereby improving the practical utility of liquid-stored boar semen in commercial AI programs.

## MATERIALS AND METHODS

### Ethical approval

All experimental procedures involving animals were reviewed and approved by the Animal Ethics Committee of Khon Kaen University in accordance with the guidelines for animal experimentation of the National Research Council of Thailand (Approval No. 54/66).

### Study period and location

The Study was conducted from December 2022 to November 2023 in Department of Surgery and Theriogenology Laboratories, Faculty of Veterinary Medicine, Khon Kaen University, Khon Kaen, Thailand.

### Animals and semen collection

Fresh semen samples were obtained from Duroc boars (≤2 years old) maintained by a commercial swine breeding facility in Khon Kaen, Thailand. Semen was collected using the gloved-hand technique and diluted immediately with a commercial Beltsville Thawing Solution (BTS; D-MAX Gold, Landata Cobiporc, France) to a final concentration of 30 × 10^6^ sperm/mL. The diluted semen was packaged in 80 mL disposable tubes and maintained at 17°C in insulated Styrofoam containers for transport to the laboratory within 90 min of collection.

### Preparation of IDB stock and working solutions

A primary stock solution of IDB was prepared at a concentration of 100 mM in high-performance liquid chromatography grade methanol (73.8 mL/tube) and stored at −20°C. Working solutions were diluted in dimethyl sulfoxide and stored at 4°C until use.

### Experimental design

A total of 22 boars were used across two independent experiments. Semen samples were evaluated on arrival for motility and concentration. Only ejaculates exhibiting ≥70% motility were included in subsequent analyses.

#### Experiment 1: Determination of optimal IDB concentration

Thirteen ejaculates were used to evaluate six concentrations of IDB (0, 78, 156, 312, 625, and 1250 nM) added to BTS-diluted semen. Samples were stored in 15 mL polypropylene tubes at 17°C and assessed at 24-h intervals up to 120 h for total motility (TM), progressive motility (PM), viability, and plasma membrane integrity.

#### Experiment 2: Evaluation of advanced sperm parameters

Twelve ejaculates were diluted in BTS with either 0 nM (control) or the optimal IDB concentration (78 nM), and stored at 17°C for 120 h. At the end of the storage period, samples were evaluated for acrosome integrity, capacitation status, LPO (through malondialdehyde [MDA] level measurement), and sperm motility parameters using computer-assisted sperm analysis (CASA).

All handling, storage conditions, and analysis protocols were standardized across experiments.

### Sperm motility assessment

Sperm motility was evaluated using a CASA system (CEROS II; Hamilton Thorne, USA). A 100 μL semen aliquot was mixed with 50 μL of 5% fetal bovine serum in BTS and incubated at 37°C for 3 min. A 15 μL drop of this mixture was placed in a homemade chamber slide (constructed with grease-spotted coverslips). A minimum of 500 spermatozoa across five fields were analyzed at 100× magnification using a Nikon Eclipse C1 microscope (Nikon Corp., Tokyo, Japan).

### Sperm viability assessment

Viability was determined through eosin-nigrosin staining following Heinemann’s protocol [35]. A 20 μL semen aliquot was mixed with 20 μL of stain, smeared on a clean glass slide, and air-dried. A total of 300 spermatozoa were examined under a light microscope (400×; Nikon Eclipse C1), with unstained (colorless) sperm considered viable.

### Plasma membrane integrity (hypo-osmotic swelling test [HOST])

Membrane integrity was assessed using the HOST as per Jeyendran *et al*. [36]. Semen (40 μL) was incubated with 760 μL of HOST solution (150 mOsm/kg) at 37°C for 30 min. The reaction was stopped with 400 μL of 32% formaldehyde. After centrifugation (1,400 × *g*, 1 min), 300 spermatozoa were evaluated under phase-contrast microscopy (400×). Coiled tails were indicative of intact membranes.

### Acrosome integrity assessment

Acrosome integrity was assessed using fluorescein isothiocyanate-conjugated peanut agglutinin (FITC-PNA). A 20 μL semen smear was air-dried, fixed in ethanol at 25°C for 10 min, and stained with FITC-PNA (20 mg/mL) under coverslips at 4°C for 30 min. Slides were rinsed, mounted with 0.22 M 1,4-diazabicylo (2,2,2)-octane (DABCO) in 20% glycerol, and evaluated using fluorescence microscopy (Nikon Eclipse C1; 400×, B-2A filter). Three hundred sperm were classified as having intact, damaged, or absent acrosomes.

### Capacitation status (chlortetracycline [CTC] staining)

Capacitation was determined using CTC staining per Fraser and Herod [37], with minor modifications. A fresh 750 μM CTC solution was prepared in buffer (20 mM Tris, 130 mM NaCl, 5 mM cysteine). Equal volumes (45 μL) of semen and CTC solution were mixed, followed by the addition of 8 μL of 12.5% glutaraldehyde in 0.5 M Tris buffer (pH 7.4). A 2 μL drop of the final mixture was mounted with 2 μL of DABCO in 45% glycerol. Three hundred spermatozoa were observed under a fluorescence microscope at 400×, and categorized into F (uniform fluorescence), B (partial post-acrosomal fluorescence), or AR (acrosome-reacted) patterns.

### LPO (MDA assay)

LPO was quantified by measuring MDA through the thiobarbituric acid (TBA) reactive substances method described by Buege and Aust [38]. Semen was stored vertically at 4°C overnight. After removing seminal plasma, the sperm concentration was adjusted to 100 × 10^6^ sperm/mL. Ferrous sulfate (250 μL, 4 mM) was added to 1 mL of semen and incubated at 37°C for 1 h. The reaction mixture (1 mL) was combined with 2 mL of TBA-trichloroacetic acid (TCA) reagent (15% TCA, 0.375% TBA, 0.25N HCl), heated at 90°C for 15 min, cooled, and centrifuged (1500 × *g*, 15 min). Absorbance of the supernatant was measured at 532 nm using a SmartSpec™ Plus spectrophotometer (Bio-Rad, USA).

### Statistical analysis

Data were analyzed using the SPSS software (v28.0, IBM Corp., NY, USA). A randomized complete block design was employed, with boars considered as blocks to reduce individual variability [39]. Data normality was assessed using the Kolmogorov–Smirnov and Shapiro–Wilk tests. Where necessary, data were transformed using log_10_, square root, natural log, or power transformations. Two-way analysis of variance (main effects only) with Duncan’s multiple range tests was applied for comparisons among treatments and storage times. Independent sample t-tests were used for comparisons between two groups. Results are expressed as mean ± standard error of the mean, with statistical significance set at p < 0.05.

## RESULTS

### Experiment 1: Dose optimization of IDB

The effects of IDB supplementation on boar sperm quality during liquid storage are summarized in Table 1. At 120 h, TM was significantly higher in the 78 nM IDB group compared to the control (p < 0.05), while no significant differences were observed in the 156 nM and 312 nM groups relative to the control. The PM did not differ significantly across treatment groups at any time point. Sperm viability was significantly improved in all IDB-treated groups compared to the control (p < 0.05). In addition, plasma membrane integrity, assessed through the HOST, was significantly enhanced in the 78 nM, 156 nM, and 312 nM groups at 120 h (p < 0.05).

**Table 1:** Mean ± SEM values of the motility evaluated by CASA for TM, PM, viability, and HOST of the boar spermatozoa storage for 120 h with different concentrations.

Sperm parameters (%)	Treatments (nM)	Storage time (hours)				

		24	48	72	96	120
TM	0	90 ± 1.27^Aa^	86 ± 1.55^Ab^	80.4 ± 3.65^ABbc^	78.3 ± 2.92^Abc^	72.9 ± 2.76^Bc^
	78	89.3 ± 1.31^Aa^	88.7 ± 1.18^Aab^	85.9 ± 1.68^ABabc^	84.3 ± 1.51^Abc^	81.4 ± 2.22^Ac^
	156	89.3 ± 1.3^Aa^	88.8 ± 1.24^Aa^	85.6 ± 2.13^Aab^	82.3 ± 2.21^Ab^	80.2 ± 1.9^ABc^
	312	89.4 ± 0.91^Aa^	87.7 ± 1.4^Aa^	84 ± 2.14^ABab^	79.2 ± 5.23^Aab^	77.8 ± 2.6^ABb^
	625	88.9 ± 1.16^Aa^	85.7 ± 1.65^Aab^	78.3 ± 4.45^Bb^	55.7 ± 8.17^Bc^	47.5 ± 8.32^Cc^
	1250	87.7 ± 1.13^Aa^	72.9 ± 4.77^Bb^	41.3 ± 8.88^Cc^	37.9 ± 8.3^Bc^	16.6 ± 6.56^Dd^
PM	0	63.8 ± 2.56^Aa^	59.7 ± 3.36^Aab^	52.5 ± 4.47^ABbc^	47.6 ± 3.02^Ac^	39.9 ± 3.83^Ac^
	78	66.6 ± 2.74^Aa^	64.2 ± 2.95^Aa^	58.5 ± 3.43^Aab^	54.9 ± 3.42^Ab^	50.6 ± 3.25^Ab^
	156	66.2 ± 2.38^Aa^	64.5 ± 3.01^Aa^	58.9 ± 3.44^Aab^	54.5 ± 3.09^Abc^	48.1 ± 3.75^Ac^
	312	64.6 ± 2.46^Aa^	60.6 ± 2.83^Aab^	54.4 ± 3.45^ABbc^	47.7 ± 4.69^Ac^	46.7 ± 3.46^Ac^
	625	64.4 ± 2.88^Aa^	57.8 ± 3.04^Ab^	43.8 ± 4.27^Bc^	28.2 ± 5.75^Bcd^	22.2 ± 4.49^Bd^
	1250	62.2 ± 3.3^Aa^	34.4 ± 5.51^Bb^	19.5 ± 6.42^Cc^	18.2 ± 5^Bc^	5.9 ± 2.92^Cc^
Viability	0	95 ± 0.51^Aa^	92.8 ± 0.73^Ab^	90.8 ± 0.58^Bc^	89.9 ± 0.47^Ccd^	87.6 ± 0.67^Cd^
	78	95.8 ± 0.5^Aa^	93.9 ± 0.66^Ab^	93 ± 0.54^Abc^	93.4 ± 0.33^Abc^	91.3 ± 0.57^Ac^
	156	95.2 ± 0.48^Aa^	94.7 ± 0.44^Aa^	93.8 ± 0.42^Aab^	92.7 ± 0.51^ABb^	91.7 ± 0.51^Ab^
	312	95.6 ± 0.58^Aa^	94.2 ± 0.7^Aabc^	93.7 ± 0.56^Aabc^	93 ± 0.47^ABbc^	91.9 ± 0.52^Ac^
	625	95.7 ± 0.63^Aa^	94.2 ± 0.69^Aab^	93.5 ± 0.66^Abc^	93.4 ± 0.51^Abc^	91.6 ± 0.51^Ac^
	1250	95.4 ± 0.58^Aa^	93.5 ± 0.77^Ab^	92.5 ± 0.47^ABbc^	91.7 ± 0.57^Bbc^	89.6 ± 0.63^Bc^
HOST	0	65.9 ± 2.74^Aa^	58.4 ± 2.16^Bb^	52.6 ± 2.31^Bbc^	48.8 ± 1.78^Bc^	46.7 ± 1.63^Bc^
	78	69.7 ± 1.7^Aa^	67.8 ± 1.5^Aa^	61.7 ± 1.78^ABb^	60.2 ± 1.73^Ab^	60.3 ± 1.24^Ab^
	156	71.1 ± 1.65^Aa^	70.4 ± 1.23^Aa^	66.8 ± 1.21^Aab^	62.4 ± 1.85^Ab^	62.2 ± 1.3^Ab^
	312	71.9 ± 1.67^Aa^	68.2 ± 2.07^Aab^	65.3 ± 1.83^ABbc^	62.3 ± 1.09^Ac^	61.3 ± 1.84^Ac^
	625	71.5 ± 1.86^Aa^	65.6 ± 1.84^Ab^	61.3 ± 1.79^ABbc^	58.4 ± 2.15^Ac^	52.3 ± 2.3^Bd^
	1250	69.4 ± 1.58^Aa^	66.3 ± 1.94^Aab^	59.1 ± 2.76^ABb^	47.3 ± 3.73^Bc^	40.3 ± 3.35^Cc^

^A,B^Different capital letters on the same column indicate significant differences (p < 0.05, Duncan *post hoc* test), ^a,b^Different lowercase letters in the same row indicate significant differences (p < 0.05, Duncan *post hoc* test). SEM=Standard error of the mean, CASA=Computer-assisted sperm analysis, TM=Total motility, PM=Progressive motility, HOST=Hypo-osmotic swelling test

### Experiment 2: Evaluation of optimal dose on advanced sperm parameters

Based on Experiment 1, 78 nM IDB was identified as the optimal concentration and further assessed against the control. As shown in Table 2, both TM and PM were significantly higher in the IDB group than in the control at 120 h (p < 0.05). However, kinematic parameters – including amplitude of lateral head displacement, curvilinear velocity, straight-line velocity, average path velocity, linearity, and straightness did not differ significantly between the two groups.

**Table 2:** Mean ± SEM values of motility and kinematic evaluated by CASA in boar spermatozoa storage for 120 h.

Sperm parameters	Treatments (nM)	Storage time (hours)				

		24	48	72	96	120
TM (%)	0	89.8 ± 1.08^Aa^	86.3 ± 1.35^Ab^	84.4 ± 1.24^Bb^	79.7 ± 1.13^Bc^	75.6 ± 1.06^Bd^
	78	90.4 ± 0.65^Aa^	88.9 ± 0.54^Aab^	88.3 ± 0.66^Ab^	85.9 ± 0.53^Ac^	84 ± 0.8^Ac^
PM (%)	0	55.8 ± 2.9^AAa^	49.5 ± 2.89^Abc^	46.5 ± 2.31^Acd^	43 ± 1.91^Acd^	40.6 ± 2.35^Bd^
	78	56.3 ± 3.06^Aa^	53 ± 2.73^Aab^	50.8 ± 2^Ab^	48.2 ± 2.09^Abc^	46.8 ± 1.72^Ac^
VCL (μm/s)	0	130 ± 3.37^Aa^	122.8 ± 3.85^Aab^	122.3 ± 2.73^Aab^	120.4 ± 3.15^Ab^	119.3 ± 4.24^Ab^
	78	127.2 ± 3.92^Aa^	123.6 ± 3.42^Aa^	123.3 ± 2.3^Aa^	120.8 ± 2.35^Aa^	120.9 ± 2.4^Aa^
VSL (μm/s)	0	49.4 ± 2.3^A^ ^Aa^	45.5 ± 2.64^Ab^	42.9 ± 1.57^Ab^	44 ± 1.88^Ab^	43.2 ± 2.01^Ab^
	78	51.2 ± 3.01^Aa^	48.1 ± 3.03^Aa^	46 ± 1.97^Aa^	45.4 ± 2.34^Aa^	45 ± 1.72^Aa^
VAP (μm/s)	0	67.3 ± 2.21^Aa^	63.1 ± 2.51^Aa^	61.3 ± 1.46^Aa^	61.8 ± 1.7^Aa^	61.5 ± 2.44^Aa^
	78	68.1 ± 2.81^Aa^	65.1 ± 2.67^Aa^	63.5 ± 1.76^Aa^	62.4 ± 2.01^Aa^	62.5 ± 1.33^Aa^
STR (%)	0	69.4 ± 1.23^Aa^	67.6 ± 1.36^Aa^	66.6 ± 0.97^Aa^	67.1 ± 1.13^Aa^	66.8 ± 1.41^Aa^
	78	71.2 ± 1.53^Aa^	69.8 ± 1.66^Aa^	68.3 ± 1.29^Aa^	68.7 ± 1.2^Aa^	67.7 ± 1.58^Aa^
LIN (%)	0	36.2 ± 1.08^Aa^	35.2 ± 1.08^Aa^	33.8 ± 0.65^Aa^	34.9 ± 0.71^Aa^	34.9 ± 0.79^Aa^
	78	38.6 ± 1.23^Aa^	37.2 ± 1.53^Aa^	35.6 ± 1.07^Aa^	36 ± 1.18^Aa^	35.6 ± 1.41^Aa^
ALH (μm)	0	5.8 ± 0.11^Aa^	5.6 ± 0.12^Aa^	5.7 ± 0.07^Aa^	5.6 ± 0.08^Aa^	5.6 ± 0.15^Aa^
	78	5.6 ± 0.12^Aa^	5.6 ± 0.09^Aa^	5.7 ± 0.07^Aa^	5.6 ± 0.06^Aa^	5.6 ± 0.11^Aa^

^A,B^Different capital letters on the same column indicate significant differences (p < 0.05. Duncan *post hoc* test). ^a,b^Different lowercase letters in the same row indicate significant differences (p < 0.05. Duncan *post hoc* test). ALH=Amplitude of lateral head displacement, VCL=Curvilinear velocity, VSL=Straight-line velocity, VAP=Average path velocity, LIN=linearity, STR=Straightness, SEM=Standard error of the mean, CASA=Computer-assisted sperm analysis, TM=Total motility, PM=Progressive motility

**Figure 1:**
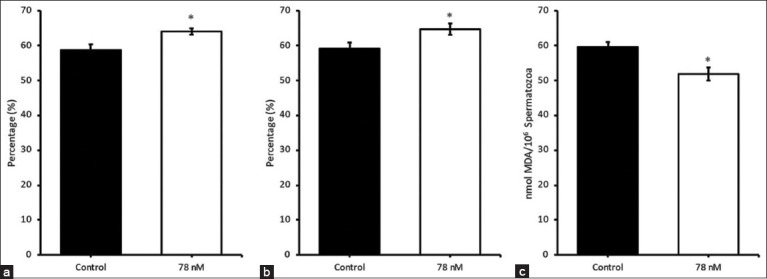
(a) Acrosome intact, (b) uncapacitated sperm, and (c) lipid peroxidation of the boar spermatozoa at 120 h of liquid storage; *Significantly different (p < 0.05) between treatments. MDA=Malondialdehyde.

Acrosome integrity analysis revealed a significantly greater percentage of intact acrosomes in the 78 nM IDB group compared to the control (p < 0.05; Figure 1a). Moreover, the proportion of capacitated spermatozoa was significantly reduced in the IDB-treated samples at 120 h (p < 0.05; Figure 1b), indicating improved functional sperm status. LPO levels, measured through MDA concentration, were also significantly lower in the IDB group than in the control, further supporting its antioxidative efficacy (p < 0.05; Figure 1c).

## DISCUSSION

### Antioxidant efficacy of *IDB* in liquid-stored boar semen

As a potent lipophilic antioxidant of the quinone family, IDB demonstrated marked efficacy in preserving boar sperm quality during liquid storage. Previous studies by Eslami *et al*. [34] and Lone *et al*. [40] have reported the beneficial effects of IDB in both liquid and cryopreserved ram semen. In this study, supplementation with 78 nM IDB significantly improved total and PM, viability, membrane integrity, acrosome integrity, and reduced LPO, highlighting its multifaceted protective role. Given the strong correlation between sperm motility and reproductive outcomes such as fertility rates and litter size in swine [41], maintaining high motility during storage is essential. Notably, CASA analysis confirmed that IDB at 78 nM sustained acceptable motility for 5 days – surpassing the conventional storage limit for BTS extenders.

### Concentration sensitivity and mitochondrial considerations

Boar spermatozoa exhibited heightened sensitivity to IDB concentration. The optimal concentration identified (78 nM or 0.078 μM) was significantly lower than that used in ram semen studies (4μM). In contrast, higher concentrations (625 nM and 1250 nM) significantly impaired motility without affecting viability or membrane integrity. This suggests a potential inhibitory effect of high-dose IDB on mitochondrial adenosine triphosphate production. Although structurally related to CoQ10, IDB can function as a complex I inhibitor [27], possibly disrupting electron transport between complexes I–II and III. Conversely, at lower concentrations, IDB may enhance mitochondrial function by promoting complex II–III electron flow [42], activating the glycerophosphate shuttle [43], or facilitating cytoplasmic electron transfer to complex III [31, 44]. Whether the motility enhancement observed here is mechanistically linked to ATP synthesis requires further investigation.

### Preservation of membrane and acrosomal integrity

Sperm membrane integrity is vital for motility, fertilization, and sperm-egg interaction. In this study, the HOST assay revealed significantly greater membrane preservation in the IDB-treated group compared to the control, consistent with findings in liquid-stored ram spermatozoa [34]. Similarly, sperm viability was enhanced across all IDB-treated groups. Acrosome integrity, a critical determinant for zona pellucida penetration and fertilization, was also significantly improved in the IDB group after 120 h of storage. These findings are consistent with a previous study by Sun *et al*. [7] that reported the protective effect of antioxidants on boar sperm acrosomes during storage.

### Inhibition of spontaneous capacitation

Spontaneous capacitation – premature capacitation in the absence of physiological inducers – can occur during semen storage and negatively impact fertilization potential [45]. In this study, IDB treatment significantly reduced the proportion of capacitated spermatozoa after 120 h. Given the established relationship between elevated ROS levels and premature capacitation [11, 46], this effect may be attributable to IDB’s antioxidant action in suppressing ROS and stabilizing the sperm plasma membrane.

### Reduction of LPO

Boar spermatozoa are highly susceptible to LPO due to their elevated PUFA content [24, 47]. MDA, a terminal product of PUFA peroxidation, is a reliable biomarker of oxidative membrane damage [48]. In agreement with the previous study by Karunakaran *et al*. [49], semen stored without anti-oxidant supplementation showed increased MDA levels over 5 days. In contrast, IDB supplementation signifi- cantly reduced MDA accumulation, indicating suppression of LPO. These results align with a previous study by Eslami *et al*. [34] on ram semen and cellular models where IDB mitigated oxidative damage [26, 50–53]. Given its benzoquinone structure, IDB can act as a direct radical scavenger by donating electrons to neutralize lipid peroxyl radicals [54].

## CONCLUSION

This study demonstrated that IDB, a mitochondrial-targeted antioxidant, significantly enhances boar sperm quality during liquid storage at 17°C for up to 120 h. The optimal concentration identified was 78 nM, which significantly improved total and PM, sperm viability, plasma membrane integrity, and acrosome integrity, while also reducing capacitation and LPO levels. Notably, the use of IDB extended the functional lifespan of sperm beyond the conventional storage limit of the BTS extender, suggesting practical benefits for AI programs in swine production.

From a practical standpoint, incorporating IDB at 78 nM into commercial semen extenders may help maintain boar semen quality over prolonged storage, potentially improving AI efficiency, fertility outcomes, and logistical flexibility for swine producers. Furthermore, this study highlights the importance of dose precision, as higher concentrations (≥625 nM) were found to adversely affect motility, possibly due to interference with mitochondrial electron transport.

The strength of this study lies in its comprehensive evaluation of both classical and advanced sperm parameters, including kinematic traits, acrosome status, capacitation, and oxidative biomarkers, under standardized storage conditions. However, some limitations must be acknowledged. The fertility outcomes associated with IDB-treated semen were not assessed in vivo, and the underlying molecular mechanisms, particularly those involving mitochondrial function and ATP production remain speculative and warrant further investigation.

Future studies should explore the *in vivo* fertilization and farrowing outcomes of IDB-supplemented semen under field conditions, as well as its potential synergistic effects with other mitochondrial antioxidants. In addition, mechanistic studies focusing on ATP production, mitochondrial membrane potential, and ROS generation dynamics will provide further insight into the bioenergetic roles of IDB in sperm physiology.

IDB at an optimal concentration of 78 nM represents a promising antioxidant additive for boar semen preservation, offering both theoretical and practical advances in swine reproductive biotechnology. Its implementation in commercial AI systems could support improved semen longevity, reduced oxidative damage, and ultimately, enhanced reproductive performance.

## DATA AVAILABILITY

All the generated data are included in the manuscript.

## AUTHORS’ CONTRIBUTIONS

RRP and SS: Compiled ideas for the experimental design and framework of research. RRP: Conducted semen processing, sperm evaluation, and statistical analysis. SS and JJ: Supervised the study and critically read and revised the manuscript for intellectual content. All authors have read and approved the final manuscript.
